# Oligoclonal Band Status in Scandinavian Multiple Sclerosis Patients Is Associated with Specific Genetic Risk Alleles

**DOI:** 10.1371/journal.pone.0058352

**Published:** 2013-03-05

**Authors:** Inger-Lise Mero, Marte W. Gustavsen, Hanne S. Sæther, Siri T. Flåm, Pål Berg-Hansen, Helle B. Søndergaard, Poul Erik H. Jensen, Tone Berge, Anja Bjølgerud, Aslaug Muggerud, Jan H. Aarseth, International Multiple Sclerosis Genetics Consortium, Kjell-Morten Myhr, Elisabeth G. Celius, Finn Sellebjerg, Jan Hillert, Lars Alfredsson, Tomas Olsson, Annette Bang Oturai, Ingrid Kockum, Benedicte A. Lie, Bettina Kulle Andreassen, Hanne F. Harbo

**Affiliations:** 1 Department of Neurology, Oslo University Hospital, Ullevål, Oslo, Norway; 2 Department of Medical Genetics, University of Oslo and Oslo University Hospital, Oslo, Norway; 3 The Danish Multiple Sclerosis Center, Department of Neurology, Copenhagen University Hospital, Rigshospitalet, Copenhagen, Denmark; 4 Institute of Basic Medical Sciences, University of Oslo, Oslo, Norway; 5 The Norwegian Multiple Sclerosis Registry and Biobank, Department of Neurology, Haukeland University Hospital, Bergen, Norway; 6 KG Jebsen Centre for MS-research, Department of Clinical Medicine, University of Bergen, Bergen, Norway; 7 Department of Clinical Neuroscience, Karolinska Institutet, The Multiple Sclerosis Research Group, Center for Molecular Medicine, Stockholm, Sweden; 8 Institute of Environmental Medicine, Karolinska Institutet, Stockholm, Sweden; 9 Department of Clinical Neuroscience, Karolinska Institutet, The Neuroimmunology Research Group, Stockholm, Sweden; 10 Department of Clinical Molecular Biology and Laboratory Sciences (EpiGen), Institute of Clinical Medicine, University of Oslo, Oslo, Norway; 11 Department of Biostiatistics, Institute of Basic Medical Sciences, University of Oslo, Oslo, Norway; 12 Institute of Clinical Medicine, University of Oslo, Oslo, Norway; Leibniz-Institute for Arteriosclerosis Research at the University Muenster, Germany

## Abstract

The presence of oligoclonal bands (OCB) in cerebrospinal fluid (CSF) is a typical finding in multiple sclerosis (MS). We applied data from Norwegian, Swedish and Danish (i.e. Scandinavian) MS patients from a genome-wide association study (GWAS) to search for genetic differences in MS relating to OCB status. GWAS data was compared in 1367 OCB positive and 161 OCB negative Scandinavian MS patients, and nine of the most associated SNPs were genotyped for replication in 3403 Scandinavian MS patients. *HLA-DRB1* genotypes were analyzed in a subset of the OCB positive (n = 2781) and OCB negative (n = 292) MS patients and compared to 890 healthy controls. Results from the genome-wide analyses showed that single nucleotide polymorphisms (SNPs) from the HLA complex and six other loci were associated to OCB status. In SNPs selected for replication, combined analyses showed genome-wide significant association for two SNPs in the HLA complex; rs3129871 (p = 5.7×10^−15^) and rs3817963 (p = 5.7×10^−10^) correlating with the HLA-DRB1*15 and the HLA-DRB1*04 alleles, respectively. We also found suggestive association to one SNP in the *Calsyntenin-2* gene (p = 8.83×10^−7^). In *HLA-DRB1* analyses HLA-DRB1*15∶01 was a stronger risk factor for OCB positive than OCB negative MS, whereas HLA-DRB1*04∶04 was associated with increased risk of OCB negative MS and reduced risk of OCB positive MS. Protective effects of HLA-DRB1*01∶01 and HLA-DRB1*07∶01 were detected in both groups. The groups were different with regard to age at onset (AAO), MS outcome measures and gender. This study confirms both shared and distinct genetic risk for MS subtypes in the Scandinavian population defined by OCB status and indicates different clinical characteristics between the groups. This suggests differences in disease mechanisms between OCB negative and OCB positive MS with implications for patient management, which need to be further studied.

## Introduction

Multiple sclerosis (MS) is an inflammatory disorder of the central nervous system leading to demyelination and axonal damage. The cause of MS is unknown, but studies support a multifactorial etiology [1]. The association of MS to genes in the major histocompatibility complex (MHC) was early established [2], and carriers of the HLA-DRB1*15∶01 allele have more than three times increased risk for the disease [3]. In recent years, genome-wide association studies (GWAS) have identified more than 50 additional non-HLA loci associated with MS susceptibility [3], [4]. However, none of the published MS GWAS have analyzed phenotypes defined by CSF findings.

The presence of OCB in CSF and not in the corresponding serum is an important diagnostic tool in MS and is thought to reflect a local B-cell response of unknown specificity and significance [5]–[8]. Up to 95% of MS patients in Northern Europe have OCB in the CSF, but this frequency varies depending on laboratory routines, study populations and was recently also related to latitude [8]–[10]. The absence of OCB in CSF has been claimed to be associated with a better, worse or equal clinical outcome compared to OCB positive MS [10]–[18]. Various findings are also reported for the genetic risk to OCB status conferred by HLA-DRB1 alleles. Several studies have shown that the HLA-DRB1*15 allele is associated with OCB positive MS [12], [18], [19] (Leone et al. personal communication), or confer a stronger risk for OCB positive MS than OCB negative MS [14], [17]. OCB negative MS has shown association to the HLA-DRB1*04∶04/04∶05 alleles [12], [19], or the HLA-DRB1*03∶01 allele and the HLA-DRB1*03∶01/*04∶01 and HLA*-*DRB1*13∶01/15∶01 genotypes [17].

On this background, we aimed to further investigate genetic differences between OCB positive and negative MS patients by using the genotype data obtained from the Scandinavian sample set (Norwegian, Swedish and Danish) included in a recent MS GWAS [3]. In a screening phase, we looked for association between genome-wide SNP data and OCB status in Scandinavian MS patients. Replication was subsequently attempted by genotyping an independent Scandinavian sample set including more than twice as many patients as the initial screening. Finally, we analyzed *HLA-DRB1* genotype data in relation to OCB status and compared with healthy controls. Data on gender, AAO, clinical course and the MS outcome measures Expanded Disability Status Scale (EDSS) and Multiple Sclerosis Severity Score (MSSS) were also available for analyses. To our knowledge, this study includes the largest sample set to date used to investigate genetic susceptibility to OCB status and detects differences in genetic risk to OCB negative and positive MS conferred mainly by HLA-DRB1 alleles.

## Materials and Methods

### Ethics Statement

The study was approved by the regional ethical committees in each country involved; The regional Committee for Medical and Health Research Ethics (REC)- South East and REC-West (Norway), the Danish Ethical Committee Review Board for Copenhagen and Frederiksberg (Denmark) and the Regional Ethical Review Board in Stockholm (Sweden). The informed written consent was obtained for all Norwegian and Danish samples included. The Swedish samples were collected in five different studies. For four of them informed written approval was obtained from the patients and controls, but for one of them only informed oral approval was obtained. The reason for this was that written approval was not mandatory at the time of collection of these samples. The procedure was such that no samples were taken unless oral approval was obtained, but no additional documentation was kept regarding the approval to participate in the study. This procedure was approved by the Regional Ethical Review Board in Stockholm.

### Patients and Controls

The screening phase initially included 1574 MS patients collected in Norway (n = 899), Denmark (n = 244) and Sweden (n = 431). In the replication phase, 3568 additional samples from Norway (n = 1236), Sweden (n = 1390) and Denmark (n = 942) underwent genotyping. All MS patients were diagnosed in accordance with the Poser and/or McDonald criteria [5], [6]. The analyses further included 428 Swedish healthy controls from the EIMS study [20] and 890 healthy controls from the Norwegian bone marrow donor registry.

### CSF Analyses

CSF analyses were performed by isoelectric focusing with immunoblotting (IEF), agarose gel electrophoresis (AGE) and/or measures of IgG index (CSF/serum IgG:CSF/serum albumin). A patient was characterized as OCB positive if oligoclonal bands were detected in the CSF by IEF or AGE and/or in the presence of increased IgG index. A patient was described as OCB negative only in the absence of OCB detection on IEF or AGE. Less than 3% of the patients had measures of IgG index only.

### Genome-wide SNP Data and SNPs for Replication

In the screening phase genotype data from 580,030 SNPs from the International Multiple Sclerosis Genetics Consortium (IMSGC) and Wellcome Trust Case Control Consortium2 (WTCCC2) GWAS was available for association analyses to OCB status in 1406 OCB positive and 168 OCB negative MS patients and 428 Swedish controls [3]. After quality control, 495,970 SNPs were left for analyses in 1528 patients (1367 OCB positive and 161 negative). All samples were genotyped by the Illumina Human 660-Quad chip performed on the Illumina Infinium platform at the Wellcome Trust Sanger Institute as described previously [3].

Nine of the top hit SNPs identified in the screening phase were selected for replication in accordance with the following criteria; p-value from the screening phase <10^−5^, level of deviation from HWE p>0.001, minor allele frequency (MAF) >5% (all SNPs but rs11790235 on chromosome 9) and the suitability of the context sequences for design of primers. GWAS cluster plots for all replicated SNPs were inspected specifically for the Scandinavian samples. Two SNPs located in the *PRKRA* gene were excluded as their context sequences also aligned to the MS associated gene *HLA-DRB1*. All the SNPs in the *HNMT* gene on chromosome 2 (n = 14) were in high or complete linkage disequilibrium (LD) (r^2^ and D’: 0.98-1). We selected three of these SNPs for genotyping from different regions (intron 1 and 3′UTR). The context sequences were obtained from UCSC Genome Browser dbSNP 132, Human Feb. 2009 assembly (http://genome.ucsc.edu/) [21].

### Genotyping

The SNPs selected for replication were genotyped by Sequenom technology (San Diego, Ca, USA). Duplicates from the screening (n = 174) and within the replication (n = 120) were genotyped as positive controls. *HLA-DRB1* genotypes of two digit resolution for 405 Norwegian patient samples were available from previous studies [22], [23], and four digit HLA typing in the remaining Norwegian patients (n = 1652) and healthy Norwegian controls (n = 890) was performed by a sequence based approach [24]. HLA typing in the Swedish samples was performed as previously described [25]. Of the Swedish samples included in our HLA-DRB1* genotype analyses, only 18 samples overlapped with the previous study by Imrell et al. [12].

### Statistical Analyses

Association testing of OCB status to the clinical parameters gender, AAO, clinical course, MS outcome measures (EDSS and MSSS) was performed by chi square testing (disease course and gender), two sample t-tests assuming equal variances (AAO) and Wilcoxon signed rank test (EDSS and MSSS). PLINK software v.1.06 (http://pngu.mgh.harvard.edu/purcell/plink/) [26] was used in quality control. In the screening, individuals with >1% missing genotypes, excess homo-/heterozygosity or who were related (pi hat <0.2) and SNPs with call rates <98% or having failed internal quality control at Sanger were excluded [3]. In the replication, individuals with >4/9 missing genotypes were excluded (n = 165). Population heterogeneity was assessed by principal component plots and genomic inflation factor λ (screening) and Breslow Day testing (replication). SNP association testing to OCB status was performed by logistic regression analyses applying an additive genotype model including AAO as a covariate. Combined analyses were performed by fixed effects meta-analyses. R 2.12.1 (www.r-project.org) was used in SNP association analyses, conditional logistic regression testing and correlation coefficient estimations. *HLA-DRB1* allelic association testing was performed in Unphased 03.01.10 where allelic frequency threshold was set to 5% [27]. The odds ratio (OR) and confidence interval (CI) for each associated *HLA-DRB1* allele were calculated by specifically testing the risk of the associated allele against all other *HLA-DRB1* alleles with frequencies above 5% pooled together.

## Results

### Genetic Association to OCB Status

Initial analyses of clinical parameters in relation to OCB status revealed nominal association of OCB status to AAO (p = 0.05) (Table 1). Hence AAO was included as a covariate in all subsequent analyses. In the genome-wide screening phase 495,970 SNPs were left for analyses in 1528 patients (1367 OCB positive and 161 negative) after quality control. In subsequent logistic regression analyses, 31 SNPs located in seven different genetic regions, including the MHC, showed a trend for association to OCB status (p<10^−5^) (Table S1). We found no evidence of population heterogeneity (λ = 1) and inclusion of the first four principal components gave comparable results (Table S1).

**Table 1:** Clinical characteristics of MS patients and association testing to OCB status.

	*Screening (n = 1574)*			*Replication (n = 3568)*			*Combined analysis (n = 5142)*		
	OCB negative (n = 168)	OCB positive (n = 1406)	p^a^	OCB negative (n = 384)	OCB positive (n = 3184)	p^a^	OCB negative (n = 552)	OCB positive (n = 4590)	p^a^
**M:F**	56 (34%):111 (66%)	395 (28%):1010 (72%)	0.14	127 (33%):257 (67%)	911 (29%):2270 (71%)	0.07	183 (33%):368 (67%)	1306 (28%):3280 (72%)	0.02
**Mean AAO**	34.7 (n = 160)	33.0 (n = 1316)	0.05	34.0 (n = 368)	33.0 (n = 3157)	0.07	34.2 (n = 528)	33.0 (n = 4473)	0.01
**PP:RR MS**	16 (10%):137 (90%)	118 (9%):1157 (91%)	0.63	38 (11%):314 (89%)	239 (9%):2414 (91%)	0.27	54 (11%):451 (89%)	357 (9%):3571 (91%)	0.24
**Mean EDSS**	2.90 (n = 97)	2.94 (n = 868)	0.86	3.63 (n = 275)	3.93 (n = 2487)	0.07	3.44 (n = 372)	3.68 (n = 3355)	0.10
**Mean MSSS**	3.85 (n = 94)	3.97 (n = 825)	0.64	4.12 (n = 271)	4.54 (n = 2427)	0.02	4.05 (n = 365)	4.40 (n = 3252)	0.02

*Abbreviations:* **M** = Male, **F** = Female, **AAO** = Age at onset, **PP** = Primary progressive, **RR** = Relapsing remitting, **OCB** = Oligoclonal bands **EDSS** = Expanded Disability Status Scale, **MSSS** = Multiple Sclerosis Severity Score.

a The p values for association between OCB status and the annotated clinical parameters.

When genotype frequencies in OCB negative and OCB positive patients were compared to Swedish healthy controls (n = 428) genotyped in the same GWAS [3] (Table S2), nearly all the identified non-HLA SNPs showed association to the OCB negative group only. On the other hand, all but two SNPs in the MHC (rs34083746 and rs3957148) showed significant association only to the OCB positive group. Rs34083746, located within *HLA-DRB1*, showed association of opposite effects in the OCB positive and OCB negative group, whereas rs3957148 near *HLA-DQA2* showed association to the OCB negative group only.

In the replication phase, nine of the 31 SNPs were analyzed in 3403 individuals (n = 3029 OCB positive and n = 374 OCB negative) (Table 2). These SNPs were selected in accordance with the criteria described in materials and methods. All SNPs had genotyping call rates above 97% and were in Hardy Weinberg equilibrium (HWE) (p>0.05) in both patient subgroups, except rs3817963 (HWE p-value = 4.8×10^−4^). In order to exclude that the deviation from HWE was due to genotyping error, we manually inspected the cluster plots of this SNP to ensure that the clusters from each genotype was distinctly separated and that no genotype appeared to have systematically dropped out. Furthermore, there were no missing genotypes in the screening phase and only 1% missing genotypes in the replication phase (32 out of 3403) for rs3817963. Since this genetic region is known to be highly disease associated, and the genotyping results appeared to be of good quality, a possible explanation for the deviation from HWE is that the genotype data arrived from cases only. Associations to OCB status were replicated for the two SNPs included from the MHC region; rs3817963 (p-value = 8.6×10^−6^, OR 1.51) and rs3129871 (p-value = 2.8×10^−10^, OR 1.69). In addition, rs17411949 in the *CLSTN2* gene on chromosome 3 showed nominal significant association (p-value 0.02, OR 1.47). No other SNP showed association in the replication phase (p>0.05). Breslow-Day testing showed no significant population heterogeneity, and adjusting for population stratification revealed comparable results (data not shown).

**Table 2:** Results of screening, replication and combined analyses of the nine SNPs brought forward to the replication.

			*Screening*			*Replication*			*Combined analysis*		
			OCB negative MS* (n = 161) OCB positive MS* (n = 1367)			OCB negative MS* (n = 374) OCB positive MS* (n = 3029)			OCB negative MS* (n = 535) OCB positive MS*(n = 4396)		
CHR	SNP	Nearest gene^a^	OR^b^	95% CI	p^c^	OR	95% CI	p^c^	OR	95% CI	p^c^
1	rs6659742	*C1ORF204*	1.82	1.43–2.32	9.1E-07	1.07	0.91–1.25	0.44	1.26	1.10–1.43	0.0007
2	rs1378321	*HNMT*	1.89	1.45–2.45	1.8E-06	1.00	0.82–1.22	0.98	1.26	1.08–1.48	0.004
2	rs4646333	*HNMT*	1.88	1.45–2.44	1.9E-06	1.02	0.84–1.25	0.82	1.28	1.09–1.49	0.002
2	rs1455158	*HNMT*	1.88	1.45–2.43	2.3E-06	1.03	0.85–1.26	0.75	1.28	1.10–1.50	0.002
3	rs17411949	*CLSTN2*	2.63	1.78–3.87	1.0E-06	1.47	1.07–2.01	0.02	1.85	1.45–2.37	8.3E-07
6	rs3817963	*BTNL2*	1.87	1.42–2.46	6.6E-06	1.51	1.26–1.80	8.6E-06	1.61	1.38–1.87	5.7E-10
6	rs3129871	*HLA-DRA*	1.79	1.40–2.29	3.9E-06	1.69	1.44–1.99	2.8E-10	1.72	1.59–1.86	5.7E-15
6	rs6926377	*UTRN*	1.80	1.40–2.33	6.4E-06	1.01	0.84–1.21	0.93	1.22	1.05–1.41	0.008
8	rs12674503	*FBXO25*	0.44	0.31–0.61	1.7E-06	1.13	0.95–1.34	0.18	0.92	0.79–1.08	0.31

* Numbers included after quality control of genotyping data.

*Abbreviations*: **CHR** = chromosome, **SNP** = single nucleotide polymorphism, **OR** = odds ratio, **CI** = confidence interval, **n** = number of individuals included in the analyses after quality control, ***HNMT*** *: histamine N-methyltransferase,* ***CLSTN2*** *= Calsyntenin-2,* ***BTLN2*** *= Butyrophilin-like protein 2,* ***UTRN*** *= Utrophin,* ***FBXO25*** *= F-box protein 25.*

a Where a SNP is located within a gene, the corresponding gene is underlined.

b The OR is given for OCB negative patients/OCB positive patients.

c The p values are calculated setting age at onset to covariate and are uncorrected for multiple testing.

Combining the results from the screening and replication phases, revealed genome-wide significant associations to OCB status for the two HLA-SNPs rs3817963 (p_combined_ = 5.7×10^−10^, OR 1.61) and rs3129871 (p_combined_ = 5.7×10^−15^, OR 1.72) and suggestive evidence that rs17411949 in the *CLSTN2* gene (p_combined_ = 8.8×10^−7^, OR 1.85) increases risk of OCB negative MS (Table 2). Combined analysis of the clinical parameters in relation to OCB status (Table 1), showed that the OCB negative patients had significantly lower MSSS (4.05 vs. 4.40, p = 0.02), older AAO (34.2 vs. 33.0, p = 0.01) and were more likely to be male (p = 0.02).

### HLA-DRB1*15∶01 and *04∶04 are Associated with OCB Status

In order to investigate for correlation of the associated SNPs in the MHC region to common *HLA-DRB1* alleles, we calculated their correlation coefficients to *HLA-DRB1* genotypes available in 822 of the 859 Norwegian samples included in the screening phase. All the HLA SNPs showed strongest correlation with either DRB1*15 or DRB1*04 alleles (r> = 0.65) (Table 3 and Table S3). We therefore analyzed the association of the HLA-SNPs in the GWAS screened Norwegian samples again, conditioning on the HLA-DRB1*15 and *04 alleles, whereby association of all HLA-SNPs became non-significant (Table 3).

**Table 3:** Conditional logistic regression and correlation analyses of the associated HLA SNPs in the screening in relation to the HLA-DRB1*15 and *04 alleles.

	*Norwegian MS samples in screening (n = 859)*	*Norwegian MS samples in screening genotyped for HLA- DRB1 (n = 822)*			
HLA SNP_allele	p-value	p^cond^ *^15^	Correlation *15	p^cond^*^04^	Correlation *04
rs2395157_G	0.008	0.07	−0.32	0.87	0.72
rs3817963_G	0.009	0.08	−0.32	0.92	0.72
rs3129871_C	0.03	0.96	−0.70	0.58	0.41
rs9268906_G	0.001	0.02	−0.36	0.49	0.80
rs34083746_G	0.001	0.02	−0.32	0.28	0.99
rs3828840_A	0.004	0.43	0.78	0.15	−0.38
rs9271640_A	0.001	0.12	0.88	0.05	−0.37
rs3129720_A	0.009	0.58	0.71	0.32	−0.41
rs9275563_A	0.01	0.08	−0.40	0.42	0.65
rs3957148_G	0.0007	0.01	−0.27	0.72	0.87

*Abbreviation*s: **HLA** = Human Leukocyte antigen, **p^cond^** = p value of the annotated HLA SNP in logistic regression analyses conditioning on the HLA-DRB1*15 (p^cond^ *^15^) or *04 (p^cond^ *^04^) alleles, correlation*15 = correlation coefficient r between annotated HLA SNP and the HLA-DRB1*15 allele, correlation*04 = correlation coefficient r between annotated HLA SNP and the HLA-DRB1*04 allele.

Since our analyses implied that associated HLA-SNPs from the screening likely reflected signals from the HLA-DRB1 *04 and *15 alleles, we performed a third stage of analyses of *HLA-DRB1* genotypes in relation to OCB status. *HLA-DRB1* genotype data with two digit resolution was available for a subset of the Norwegian (n = 2068) and Swedish MS patients (n = 1005) (including in total 292 OCB negative patients). When comparing *HLA-DRB1* allele frequencies in OCB negative and OCB positive MS patients, HLA-DRB1*15 conferred relative protection for OCB negative MS (p-value = 1.28×10^−7^, OR 0.59, 95% CI 0.48–0.73) and HLA-DRB1*04 increased risk of OCB negative MS compared with OCB positive MS (p-value = 1.46×10^−7^, OR 1.80, 95% CI = 1.44–2.25) (data not shown).

To refine the *HLA-DRB1* association, we used *HLA-DRB1* genotypes of four-digit resolution available for 1652 of the Norwegian MS samples and 890 healthy Norwegian controls (Table 4). Comparing OCB negative (n = 199) and OCB positive (n = 1453) MS patients showed that HLA-DRB1*15∶01 and HLA-DRB1*04∶04 were significantly differently distributed between OCB positive and OCB negative MS patients (p_*15∶01_ = 9.9×10^−5^, OR = 0.64, 95% CI 0.49–0.83, p_*04∶04_ = 0.0004, OR = 2.2, 95% CI = 1.5–3.20). As expected, the HLA-DRB1*15∶01 subtype accounted for the majority of the HLA-DRB1*15 allele group (99,8% in the OCB positive patients and 100% in the OCB negative patients). On the other hand, the HLA-DRB1*04∶04 subtype accounted for a larger proportion of DRB1*04 among the OCB negative patients (43,7%) than the OCB positive (34,1%).

**Table 4:** Subtypes of HLA-DRB1 alleles showing association to OCB positive and/or OCB negative MS in Norwegian MS patients and healthy controls.

	Allele frequencies (%)^a^			OCB negative MS vs. OCB positive MS		OCB positive MS vs. controls			OCB negative MS vs. controls		
HLA-DRB1* allele	OCB− n = 199	OCB+ n = 1453	Controls n = 890	OR (95% CI)	p^b^	OR (95% CI)	p^b^	p^c (^*^1501)^	OR (95% CI)	p^b^	p^c (^*^1501)^
*15∶01	25.1	34	15.1	0.64 (0.49–0.83)	9.9E-05	2. 94 (2.50–3.46)	2.6E-53		1.88 (1.41–2.50)	1.5E-05	
*04∶04	11.3	5.7	7.2	2.2 (1.50–3.20)	0.0004	0.71 (0.56–0.91)	0.04	0.74	1.58 (1.09–2.30)	0.02	0.01
*04∶01	11.1	8.8	12	1.33 (0.94–1.89)	0.18	0.65 (0.53–0.80)	0.0005	0.32	0.87 (0.61–1.24)	0.60	0.48
*01∶01	4.8	5.4	10.3	0.90 (0.55–1.47)	0.60	0.45 (0.36–0.57)	6.9E-10	0.03	0.41 (0.25–0.66)	2.0E-05	0.02
*03∶01	14.1	13.8	13	1.06	0.87	1.0 (0.84–1.19)	0.41	0.0006	1.05 (0.77–1.43)	0.59	0.20
*07∶01	6.3	5.7	8.9	1.13 (0.70–1.81 )	0.67	0.57 (0.45–0.72)	5.9E-05	0.04	0.65 (0.40–1.04)	0.05	0.02
*13∶01	5.0	5.9	7.8	0.86 (0.54–1.37)	0.49	0.69 (0.54–0.87)	0.01	0.85	0.59 (0.37–0.94)	0.04	0.12

*Abbreviations*: **n** = total Norwegian samples available with HLA-DRB1* genotypes of 4 digit solution, i.e. MS patients from the screening and replication are pooled, **OCB** = oligoclonal bands, **OR** = odds ratio, **CI** = confidence interval.

a The results are only shown for alleles with frequencies >5%.

b All p values are uncorrected for multiple testing.

c ( *^1501)^ p values after stratification for HLA-DRB1*15∶01.

### Shared Effects at the HLA-DRB1*15∶01, *01∶01, *07∶01 Loci and Opposite Effects at the HLA-DRB1*04∶04 Allele

**Figure 1:**
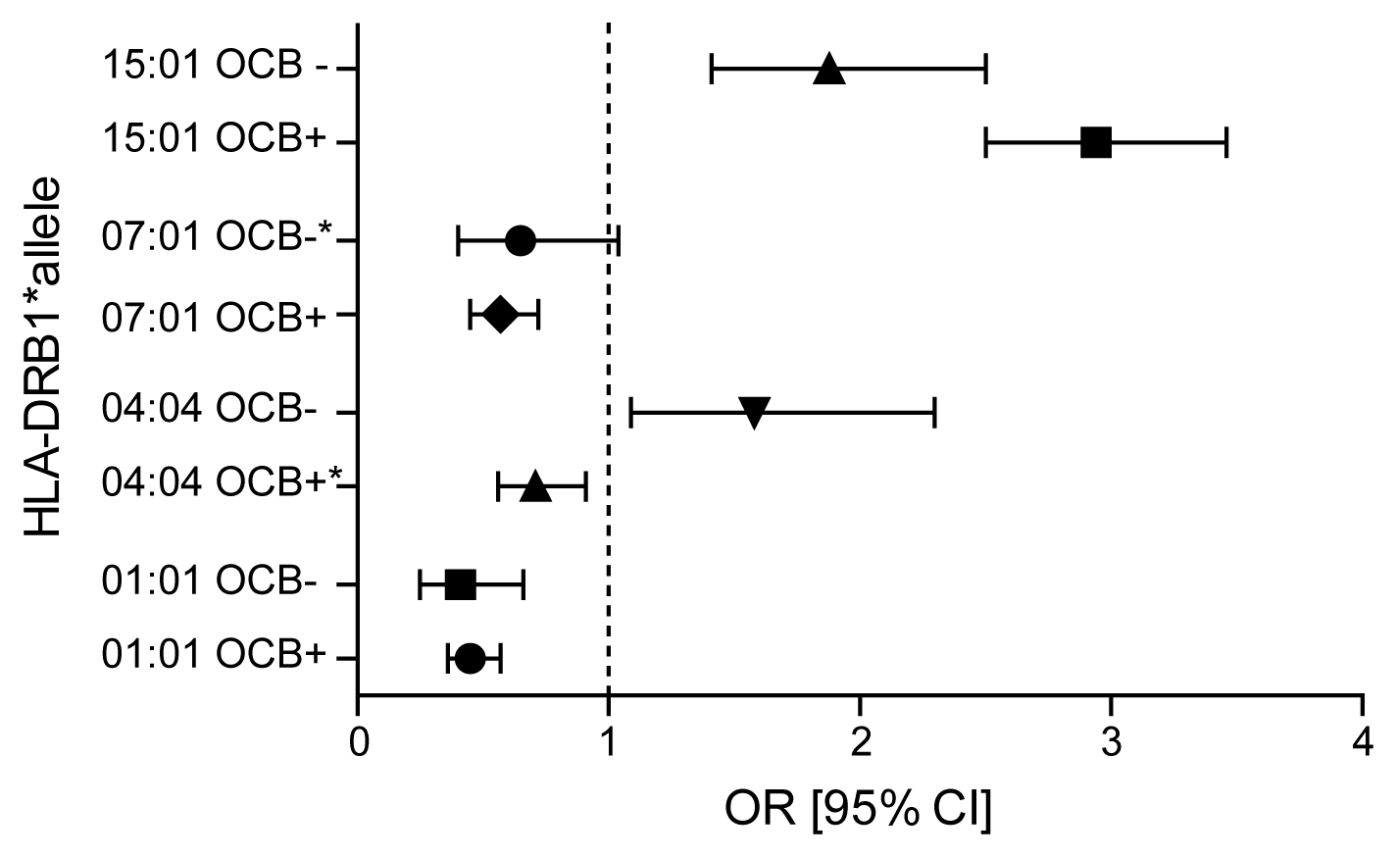
HLA-DRB1 alleles with significant effects on OCB positive and negative MS. Figure 1 shows the ORs and 95% CI for *HLA-DRB1* alleles with significant effects on OCB positive and negative MS. Only alleles which remain significant after stratification for HLA-DRB1*15∶01 allele in both groups are included, except HLA-DRB1*04∶04 (not withstanding stratification in the OCB positive group) and HLA-DRB1* 07∶01 (associated to OCB negative MS only after stratification). These exceptions are marked (*).

Compared to healthy controls the HLA-DRB1*15∶01 was a risk allele for both CSF subtypes, but conferred a stronger risk on OCB positive than OCB negative MS (OCB positive; p-value = 2.57×10^−53^, OR = 2.94, OCB negative; p-value = 1.53×10^−5^, OR = 1.88). On the other hand, HLA-DRB1*04∶04 was seen to increase risk of OCB negative MS (p-value = 0.02, OR = 1.58), but confer protection to OCB positive MS (p-value = 0.04, OR 0.71). HLA-DRB1*01∶01 and HLA-DRB1*07∶01 and HLA-DRB1*13∶01 alleles were seen to reduce the risk of both OCB negative and OCB positive MS (OCB positive; p_*01∶01_ = 7×10^−10^, OR 0.45, p_*07∶01_ = 6×10^−5^, OR 0.57, p_*13∶01_ = 0.01, OR 0.69, OCB negative; p_*01∶01_ = 2×10^−5^, OR 0.41, p_*07∶01_ = 0.05, OR 0.65, p_*13∶01_ = 0.04, OR 0.59). DRB1*04∶01 was associated with protection for OCB positive MS only (p-value = 0.0005, OR = 0.65) (Figure 1).

Since the HLA-DRB1*15∶01 allele appeared as a strong risk allele skewing the overall allelic distribution at HLA-DRB1, we next analyzed the association of the other DRB1 alleles after stratification for DRB1*15∶01 to test if these associations represented real risk effects. The association between OCB negative MS and HLA-DRB1*04∶04 remained significant (p-value = 0.01), but became non-significant in the OCB positive group (p-value = 0.74) (Table 4). Associations to HLA-DRB1*01∶01 and *07∶01 were reduced, but still nominally significant, whereas the associations detected at the *13∶01 and *04∶01 alleles were non-significant.

Association of HLA-DRB1*03∶01 (p-value = 0.0006, OR 1.5) to OCB positive MS was revealed. When looking at the genotype distribution, the DRB1*03∶01 association could be explained by excess homozygosity of HLA-DRB1*03∶01 in OCB positive patients (frequency = 3.3%) compared to controls (frequency = 1.4%), leading to a higher frequency of the HLA-DRB1*03∶01 allele in the patients. The OCB negative group also showed higher frequency of HLA-DRB1*03∶01 homozygotes (frequency = 4%) compared to controls.

## Discussion

This study reports the results of a genome-wide screening and subsequent replication of a genotype-OCB phenotype analysis in a total of 4931 genetically homogenous Scandinavian MS patients. In all stages of the study, we found strong evidence that genetic differences relating to OCB status of Scandinavian MS patients could be exerted by HLA class II loci. All HLA associated SNPs in the screening phase were located in or near HLA class II genes. Further, high correlation coefficients were estimated between the top hit HLA SNPs in the screening and the HLA-DRB1*15 and *04 alleles. Accordingly, we found no evidence of association in Norwegian samples included in the screening to any of the identified HLA-SNPs when association to HLA-DRB1*15 or *04 alleles were accounted for in conditional logistic regression analyses. Of note, the SNP used to tag HLA-DRB1*15∶01 in the MS GWAS (rs3129934) [3] yielded a p-value of 5.13×10^−5^ in the screening phase (data not shown), supporting association of HLA-DRB1*15∶01 to OCB status. Analyses of *HLA-DRB1* alleles indeed showed that OCB positive and OCB negative MS differ by their association to HLA-DRB1*15. Moreover, HLA-DRB1*15∶01 was a common risk factor for both OCB negative and OCB positive MS, but exerted a stronger effect on OCB positive MS. On the other hand, the HLA-DRB1*04∶04 allele showed opposite effects in the two patients groups, i.e. increasing risk of OCB negative MS and somewhat protecting against OCB positive MS. These results confirm the findings from a previous, smaller Swedish study [12].

In addition, we showed that HLA-DRB1*01∶01 and *07∶01 reduce risk of OCB negative and OCB positive MS. Protective effects of HLA-DRB1*07 and *01∶01 to MS have previously been reported [25], [28], [29]. HLA-DRB1*03∶01 showed association to OCB positive MS after stratification for HLA-DRB1*15∶01 and higher frequencies of homozygotes for the HLA-DRB1* 03∶01 allele were seen in both OCB negative and OCB positive MS compared to controls. This complies with an earlier reported recessive risk for MS conferred by the HLA-DRB1*03 allele [30].

Our data also strongly suggested that rs17411949 in intron 1 of the *CLSTN2* gene at chromosome 3 increased the risk of OCB negative MS. Variants of this gene have been reported to be associated with memory and late onset Alzheimer’s disease in GWASs, but with inconsistent replication [31]–[33]. CLSTN2 is predominantly expressed in the brain and is localized in the postsynaptic membrane of excitatory synapses where it binds synaptic Ca^2+^ [34]. However, CLSTN2 is also expressed in bone marrow, tonsils and Daudi cells (B cell lymphoma) (http://genome.ucs.edu/), and might have an immunological function. Further analyses are required to conclude on the indicated association of this SNP in OCB negative MS.

The relatively low number of OCB negative patients in the screening phase represents a natural limitation in this study, since only approximately 10% of MS patients are negative for OCB. Nevertheless, this study sample is probably the largest available to date investigating OCB phenotype. Power calculations showed that with our sample size in the screening phase, the effect size would need to be OR >2 in order to have 80% power to detect differences between OCB positive and OCB negative MS at a significance level of p<10^−5^ (http://ihg2.helmholtz-muenchen.de/cgi-bin/hw/power2.pl). Hence the p<10^−5^ cut off in the screening phase could mean that we missed true association of ORs <2. A recent study of a German and Belgian MS patient population reported genome-wide significant association between quantitative IgG index and a genetic region different from the findings described in our study, i.e. the *IGHC* locus [35]. However, the results of these two studies are not conflicting but complementary, due to the analysis of differently defined CSF parameters. Hence, the observed CSF phenotype-genotype associations in our and the German-Belgian study probably have different biological implications.

A possible confounding factor in our study is that different methods were used for OCB detection since sample collection has been ongoing through many years. The most sensitive method for OCB detection is IEF [8]. Samples which were collected prior to the introduction of this method, were investigated by less sensitive methods, i.e. AGE or IgG index. Since AGE, which was used to annotate OCB negative MS, is less sensitive than IEF, some of the earlier collected samples may have been falsely regarded as OCB negative. However, this would in worst case deflate the association seen to the OCB negative group, rather than create falsely positive association, and hence it should not invalidate our positive findings.

Clinical studies support differences in the outcome in OCB positive and OCB negative patient groups, albeit with contrasting findings. In our combined clinical analysis of this Scandinavian dataset, we find a significantly older AAO in the OCB negative patients (p-value = 0.01), a trend previously indicated in other studies [12], [16]. A recent study validated this finding and showed a small, but very significant effect (OR 0.98, p<0.0001) of OCB status on AAO [10]. The older AAO in the OCB negative group also speaks against false inclusion of OCB negative patients caused by lumbar punctures performed in early stages of disease, when the rate of OCB detection is reported to be lower [36]. We also found a significantly lower MSSS among the OCB negative patients included in the replication analysis and the combined analysis (p-value = 0.02). This is in accordance with most studies of clinical differences related to OCB status, which suggest a better outcome for MS patients without OCB, as measured by lower degree of disability, lower frequencies of exacerbations and slower progression [10], [11], [13], [15]. On the other hand, a recent Canadian study in 1120 MS patients did not find any relation between OCB status and disease progression [37]. The conflicting findings concerning OCB status and clinical outcome, require further studies. Finally, therapeutic studies have reported that OCB negative patients are less likely to develop neutralizing antibodies to interferon-beta treatment and show better clinical response to this treatment than their OCB positive counterparts [38], [39].

OCB status and the frequency of the HLA-DRB1*15∶01 allele have been shown to vary with latitude [10], [40]. In countries like Sardinia, Japan and Turkey where about five times higher ratios of OCB negative/OCB positive MS are reported compared to Northern Europe, MS risk has been shown to be conferred by both HLA-DRB1*04 and HLA-DRB1*15∶01 [10], [12], [19], [41], [42]. Hence, we speculate that the HLA-DRB1*15∶01 allele possibly interacts with environmental agents in areas where this allele is common, making a population more prone to OCB positive MS. The HLA-DRB1*04 allele may, on the other hand, confer risk of OCB negative MS by different immuno-genetic mechanisms.

In conclusion, this study points to both distinct and shared genetic risk factors in Scandinavian MS patients differing by the presence or absence of OCB in CSF and reveals clinical differences between the groups. Risk conferred by HLA-DRB1*15∶01 was present in both OCB positive and negative groups, but was much stronger in the OCB positive group. HLA-DRB1*04∶04 conferred risk for OCB negative MS and protection of OCB positive MS. HLA-DRB1*01∶01 and *07∶01 conferred protection irrespective of OCB status. Moreover, a SNP in the *CLSTN2* gene was associated with increased risk of OCB negative MS, but requires validation in further studies. The OCB negative patients had a significantly lower MSSS, were more likely to be male and had a later age of MS onset. The identified genetic and clinical differences relating to OCB status in MS may reflect variation in disease mechanisms and possibly be of importance for patient management.

## Supporting Information

Table S1 SNPs showing association in the screening phase to OCB status of MS (p<10^−5^).
(DOCX)

Table S2 Analyses of the top hit SNPs in the screening phase comparing OCB positive and OCB negative MS patients to healthy controls.
(DOCX)

Table S3 The correlation coefficient r between the associated SNPs in the HLA region and common HLA-DRB1 alleles.
(DOCX)

File S1 List of the principle investigators in the International Multiple Sclerosis Genetic Consortium (IMSGC).
(DOCX)
