# Supplementary material for: Oligoclonal Band Status in Scandinavian Multiple Sclerosis Patients Is Associated with Specific Genetic Risk Alleles
Source: PLoS One. 2013 Mar 5;8(3):e58352. doi: 10.1371/journal.pone.0058352 (PMC3589422; doi:10.1371/journal.pone.0058352)
Supplement: Table S1 — SNPs showing association in the screening phase to OCB status of MS (p<10−5). (DOCX) [file pone.0058352.s001.docx]

**Table S1: SNPs showing association in the screening phase to OCB status of MS (p<10^-5^)**

| **CHR** | **SNP^a^** | **Nearest gene^b^** | **Genotypes** | **OCB negative MS genotypes n=161** | **OCB positive MS genotypes n=1367** | **p^HWE^** | **OR^c^** | **95% CI** | **p^1 d^** | **p^2 e^** |
| --- | --- | --- | --- | --- | --- | --- | --- | --- | --- | --- |
| **1** | **rs6659742** | ***C1ORF204*** | **AA/AG/GG** | **42/83/36** | **203/643/520** | **0.75** | **1.82** | **1.43-2.32** | **9.1E-07** | **9.5E-07** |
| 2 | rs1455167 | *HNMT* | AA/AC/CC | 13/74/74 | 59/394/913 | 0.09 | 1.88 | 1.45-2.44 | 2.1E-06 | 1.1E-06 |
| 2 | rs2737385 | *HNMT* | CC/AC/AA | 13/74/74 | 59/393/915 | 0.09 | 1.89 | 1.45-2.45 | 1.8E-06 | 9.4E-07 |
| 2 | rs3828168 | *HNMT* | AA/AG/GG | 13/74/74 | 59/391/916 | 0.08 | 1.89 | 1.46-2.45 | 1.8E-06 | 8.8E-07 |
| **2** | **rs1378321** | ***HNMT*** | **GG/AG/AA** | **13/74/74** | **59/393/915** | **0.09** | **1.89** | **1.45-2.45** | **1.6E-06** | **9.4E-07** |
| 2 | rs1455164 | *HNMT* | GG/AG/AA | 13/74/74 | 59/396/911 | 0.11 | 1.87 | 1.44-2.43 | 2.4E-06 | 1.3E-06 |
| 2 | rs993891 | *HNMT* | AA/AC/CC | 13/74/74 | 59/397/911 | 0.11 | 1.87 | 1.44-2.43 | 2.6E-06 | 1.4E-06 |
| 2 | rs2604460 | *HNMT* | GG/GC/CC | 13/74/74 | 58/399/907 | 0.15 | 1.87 | 1.44-2.43 | 2.7E-06 | 1.4E-06 |
| 2 | rs1455159 | *HNMT* | CC/AC/AA | 13/74/74 | 59/394/913 | 0.09 | 1.88 | 1.45-2.44 | 2.1E-06 | 1.1E-06 |
| 2 | rs2198652 | *HNMT* | AA/AG/GG | 13/73/74 | 59/397/911 | 0.11 | 1.86 | 1.43-2.41 | 3.5E-06 | 1.1E-06 |
| 2 | rs4245861 | *HNMT* | AA/AG/GG | 13/74/74 | 59/393/914 | 0.09 | 1.88 | 1.45-2.44 | 1.9E-06 | 9.9E-07 |
| **2** | **rs4646333** | ***HNMT*** | **AA/AG/GG** | **13/74/74** | **59/393/914** | **0.09** | **1.88** | **1.45-2.44** | **1.9E-06** | **1.0E-06** |
| **2** | **rs1455158** | ***HNMT*** | **AA/AG/GG** | **13/74/74** | **59/395/912** | **0.09** | **1.88** | **1.45-2.43** | **2.3E-06** | **1.2E-06** |
| 2 | rs1455157 | *HNMT* | GG/AG/AA | 13/74/74 | 59/396/910 | 0.11 | 1.87 | 1.44-2.43 | 2.6E-06 | 1.4E-06 |
| 2 | rs1455156 | *HNMT* | GG/GC/CC | 13/73/75 | 59/395/913 | 0.09 | 1.85 | 1.42-2.40 | 4.1E-06 | 2.2E-06 |
| 2 | rs9283487 | PRKRA^f^ | GG/AG/AA | 11/81/67 | 36/486/830 | 0.0001 | 2.15 | 1.62-2.86 | 1.2E-07 | 1.9E-08 |
| 2 | rs3997876 | PRKRA^f^ | AA/AG/GG | 13/80/66 | 39/485/831 | 0.001 | 2.19 | 1.66-2.89 | 3.3E-08 | 4.6E-09 |
| **3** | **rs17411949** | ***CLSTN2*** | AA/AG/GG | **3/30/128** | **6/112/1249** | **0.03** | **2.63** | **1.78-3.87** | **1.0E-06** | **1.9E-07** |
| 6 | rs2395157 | *BTNL2* | GG/AG/AA | 12/76/73 | 45/474/848 | 0.02 | 1.89 | 1.44-2.49 | 4.4E-06 | 7.6E-06 |
| **6** | **rs3817963** | ***BTNL2*** | **GG/AG/AA** | **12/76/73** | **46/478/843** | **0.02** | **1.87** | **1.42-2.46** | **6.6E-06** | **4.9E-06** |
| **6** | **rs3129871** | ***HLA-DRA*** | **CC/AC/AA** | **64/74/23** | **321/673/373** | **0.38** | **1.79** | **1.40-2.29** | **3.9E-06** | **2.1E-05** |
| 6 | rs9268906 | *HLA-DRA* | GG/AG/AA | 13/80/68 | 47/493/822 | 0.01 | 2.01 | 1.53-2.65 | 5.2E-07 | 9.2E-08 |
| 6 | rs34083746 | HLA-DRB1 | GG/AG/AA | 5/74/80 | 11/380/964 | 3.53E-06 | 2.45 | 1.80-3.35 | 1.6E-08 | 3.0E-09 |
| 6 | rs3828840 | *HLA-DRB1* | AA/AG/GG | 20/67/74 | 287/703/372 | 0.47 | 0.55 | 0.42-0.71 | 4.6E-06 | 2.1E-05 |
| 6 | rs9271640 | *HLA-DQA1* | AA/AG/GG | 15/55/90 | 221/674/464 | 0.91 | 0.52 | 0.40-0.68 | 2.1E-06 | 8.8E-06 |
| 6 | rs3129720 | *HLA-DQB1* | AA/AG/GG | 24/58/79 | 321/665/372 | 0.09 | 0.54 | 0.42-0.70 | 1.3E-06 | 6.6E-06 |
| 6 | rs9275563 | *HLA-DQA2* | AA/AG/GG | 24/79/54 | 88/590/676 | 0.02 | 1.90 | 1.46-2.46 | 1.6E-06 | 4.6E-07 |
| 6 | rs3957148 | *HLA-DQA2* | GG/AG/AA | 4/62/95 | 9/312/1046 | 0.002 | 2.27 | 1.65-3.13 | 4.5E-07 | 1.5E-07 |
| **6** | **rs6926377** | ***UTRN*** | **CC/AC/AA** | **19/70/72** | **72/487/808** | **0.83** | **1.80** | **1.40-2.33** | **6.4E-06** | **8.6E-06** |
| **8** | **rs12674503** | ***FBXO25*** | **AA/AC/CC** | **4/38/119** | **94/541/732** | **1.00** | **0.44** | **0.31-0.61** | **1.7E-06** | **2.0E-06** |
| 9 | rs11790235 | no gene (200kb) | AA/AG/GG | 0/37/124 | 2/140/1225 | 0.17 | 2.53 | 1.69-3.80 | 7.6E-06 | 8.9E-06 |

*Abbreviations*: **CHR** = chromosome, **SNP** = single nucleotide polymorphism, **OR** = odds ratio, **CI** = confidence interval, **p^HWE^** = p value for deviation from Hardy Weinberg equilibrium. ***HNMT*** = *histamine N-methyltransferase*, ***PRKRA*** = *Protein kinase, interferon-inducible double stranded RNA dependent activator*, ***CLSTN****2* = *Calsyntenin 2*, ***BTLN2*** = *Butyrophilin-like protein 2*, ***UTRN*** = *Utrophin*, ***FBXO25*** = *F-box protein 25*.

^a^The nine SNPs selected for replication are shown in bold.

^b^Where a SNP is located in a gene, the corresponding gene name is underlined.

^c^The odds ratio is given for OCB negative patients/OCB positive patients.

^d^p^1^ = p value adjusted for age at onset.

^e^p^2^ = p value adjusted for age at onset and first four principal components.

^f^The context sequences of these SNPs also align to DRB1 at chr6_ssto_hap7.
