# Supplementary material for: Oligoclonal Band Status in Scandinavian Multiple Sclerosis Patients Is Associated with Specific Genetic Risk Alleles
Source: PLoS One. 2013 Mar 5;8(3):e58352. doi: 10.1371/journal.pone.0058352 (PMC3589422; doi:10.1371/journal.pone.0058352)
Supplement: Table S2 — Analyses of the top hit SNPs in the screening phase comparing OCB positive and OCB negative MS patients to healthy controls. (DOCX) [file pone.0058352.s002.docx]

**Table S2: Analyses of the top hit SNPs in the screening comparing OCB positive and OCB negative MS patients to healthy controls**

|  |  |  | *CSF positive MS(n=1367) versus healthy controls (n=428)^c^* | | | *CSF negative MS( n=161) versus healthy controls (n=428)^c^* | | |
| --- | --- | --- | --- | --- | --- | --- | --- | --- |
| **CHR** | **SNP^a^** | **Nearest gene^b^** | **OR** | **95% CI** | **p** | **OR** | **95% CI** | **p** |
| **1** | **rs6659742** | ***C1ORF204*** | **1.12** | **0.96-1.32** | **0.16** | **1.99** | **1. 52-2.61** | **6.9E-07** |
| 2 | rs1455167 | *HNMT* | 0.93 | 0.77-1.13 | 0.46 | 1.85 | 1.37-2.50 | 5.7E-05 |
| 2 | rs2737385 | *HNMT* | 0.92 | 0.76-1.11 | 0.37 | 1.83 | 1.36-2.48 | 7.4E-05 |
| 2 | rs3828168 | *HNMT* | 0.91 | 0.76-1.10 | 0.35 | 1.83 | 1.36-2.48 | 7.4E-05 |
| **2** | **rs1378321** | ***HNMT*** | **0.92** | 0.76-1.11 | **0.39** | **1.84** | 1.36-2.49 | **6.7E-05** |
| 2 | rs1455164 | *HNMT* | 0.92 | 0.76-1.12 | 0.41 | 1.83 | 1.36-2.48 | 7.4E-05 |
| 2 | rs993891 | *HNMT* | 0.93 | 0.77-1.12 | 0.45 | 1.84 | 1.36-2.49 | 6.7E-05 |
| 2 | rs2604460 | *HNMT* | 0.93 | 0.77-1.12 | 0.44 | 1.83 | 1.36-2.48 | 7.4E-05 |
| 2 | rs1455159 | *HNMT* | 0.93 | 0.77-1.12 | 0.42 | 1.84 | 1.36-2.49 | 6.6E-05 |
| 2 | rs2198652 | *HNMT* | 0.93 | 0.77-1.12 | 0.42 | 1.82 | 1.35-2.46 | 9.2E-05 |
| 2 | rs4245861 | *HNMT* | 0.93 | 0.77-1.13 | 0.49 | 1.88 | 1.39-2.54 | 4.4E-05 |
| **2** | **rs4646333** | ***HNMT*** | **0.92** | 0.76-1.12 | **0.42** | **1.85** | **1.37-2.49** | **6.2E-05** |
| **2** | **rs1455158** | ***HNMT*** | **0.93** | 0.77-1.12 | **0.44** | **1.85** | **1.37-2.49** | **6.2E-05** |
| 2 | rs1455157 | *HNMT* | 0.93 | 0.77-1.13 | 0.46 | 1.85 | 1.37-2.49 | 6.2E-05 |
| 2 | rs1455156 | *HNMT* | 0.93 | 0.77-1.12 | 0.45 | 1.82 | 1.35-2.45 | 9.6E-05 |
| 2 | rs9283487 | PRKRA | 0.63 | 0.53-0.76 | 1.0E-06 | 1.20 | 0.91-1.59 | 0.21 |
| 2 | rs3997876 | PRKRA | 0.62 | 0.52-0.75 | 2.7E-07 | 1.21 | 0.92-1.59 | 0.17 |
| **3** | **rs17411949** | ***CLSTN2*** | **1.11** | **0.76-1.61** | **0.59** | **2.84** | **1.75-4.62** | **2.6E-05** |
| 6 | rs2395157 | *BTNL2* | 0.66 | 0.55-0.79 | 7.6E-06 | 1.16 | 0.88-1.53 | 0.30 |
| **6** | **rs3817963** | ***BTNL2*** | **0.66** | 0.56-0.79 | **7.6E-06** | **1.15** | **0.87-1.51** | **0.33** |
| **6** | **rs3129871** | ***HLA-DRA*** | **1.98** | **1.68-2.32** | **1.0E-16** | **1.10** | **0.84-1.43** | **0.48** |
| 6 | rs9268906 | *HLA-DRA* | 0.61 | 0.51-0.73 | 5.3E-08 | 1.11 | 0.85-1.46 | 0.46 |
| 6 | rs34083746 | HLA-DRB1 | 0.67 | 0.54-0.82 | 0.0001 | 1.44 | 1.06-1.95 | 0.02 |
| 6 | rs3828840 | *HLA-DRB1* | 1.94 | 1.64-2.29 | 5.4E-15 | 1.08 | 0.84-1.41 | 0.54 |
| 6 | rs9271640 | *HLA-DQA1* | 2.37 | 1.97-2.84 | 2.2E-20 | 1.20 | 0.90-1.60 | 0.21 |
| 6 | rs3129720 | *HLA-DQB1* | 1.91 | 1.62-2.24 | 4.9E-15 | 1.04 | 0.82-1.33 | 0.74 |
| 6 | rs9275563 | *HLA-DQA2* | 0.57 | 0.49-0.68 | 6.3E-11 | 0.99 | 0.77-1.28 | 0.96 |
| 6 | rs3957148 | *HLA-DQA2* | 0.81 | 0.64-1.02 | 0.07 | 1.70 | 1.21-2.38 | 0.002 |
| **6** | **rs6926377** | **UTRN** | **0.76** | **0.63-0.90** | **0.002** | **1.29** | **0.97-1.70** | **0.08** |
| **8** | **rs12674503** | **FBXO25** | **1.29** | **1.07-1.55** | **0.007** | **0.56** | **0.39-0.81** | **0.002** |
| 9 | rs11790235 | no gene (200kb) | 1.00 | 0.71-1.41 | 1.00 | 2.35 | 1.48-3.74 | 0.0003 |

*Abbreviations*: *CHR* = chromosome, **SNP** = single nucleotide polymorphism, n = total individuals with genotypes whom passed quality control, **OR** = odds ratio, **CI**= confidence interval. ***HNMT*** *= histamine N-methyltransferase*, ***PRKRA*** = *Protein kinase, interferon-inducible double stranded RNA dependent activator*, ***CLSTN2*** = *Calsyntenin 2*, ***BTLN2*** = *Butyrophilin-like protein 2*, ***UTRN*** = *Utrophin*, ***FBXO25*** = *F-box protein 25*.

^a^The SNPs brought forward to replication are shown in bold.

^b^Where a SNP is located in a gene, the corresponding gene name is underlined.

^c^The tested samples included all Scandinavian patients in the screening and healthy Swedish controls genotyped by the same methods.
