# Supplementary material for: Oligoclonal Band Status in Scandinavian Multiple Sclerosis Patients Is Associated with Specific Genetic Risk Alleles
Source: PLoS One. 2013 Mar 5;8(3):e58352. doi: 10.1371/journal.pone.0058352 (PMC3589422; doi:10.1371/journal.pone.0058352)
Supplement: Table S3 — The correlation coefficient r between the associated SNPs in the HLA region and common HLA-DRB1 alleles. (DOCX) [file pone.0058352.s003.docx]

**Table S3: The correlation coefficient r between the associated SNPs in the HLA region and common HLA-DRB1 alleles**

|  | *Correlation to individual HLA-DRB1*alleles ( n= 822 Norwegian samples)* | | | | | |
| --- | --- | --- | --- | --- | --- | --- |
| **HLA SNP_allele** | ***15** | ***04** | ***03** | ***13** | ***01** | ***07** |
| rs2395157_G | -0.32 | 0.72 | -0.27 | 0.02 | -0.10 | 0.29 |
| rs3817963_G | -0.32 | 0.72 | -0.27 | 0.02 | -0.10 | 0.29 |
| rs3129871_C | -0.70 | 0.41 | 0.38 | 0.10 | 0.28 | -0.19 |
| rs9268906_G | -0.36 | 0.80 | -0.28 | -0.17 | -0.02 | 0.47 |
| rs34083746_G | -0.32 | 0.99 | -0.21 | -0.14 | -0.01 | -0.06 |
| rs3828840_A | 0.78 | -0.38 | -0.33 | -0.31 | 0.22 | -0.17 |
| rs9271640_A | 0.88 | -0.37 | -0.26 | -0.30 | -0.31 | -0.13 |
| rs3129720_A | 0.71 | -0.41 | -0.37 | 0.37 | -0.16 | -0.15 |
| rs9275563_A | -0.40 | 0.65 | -0.32 | -0.16 | -0.06 | 0.40 |
| rs3957148_G | -0.27 | 0.87 | -0.17 | -0.12 | -0.03 | -0.07 |

*Abbreviations:* HLA = Human leukocyte antigen, SNP = single nucleotide polymorphism
