# Supplementary material for: Oligoclonal Band Status in Scandinavian Multiple Sclerosis Patients Is Associated with Specific Genetic Risk Alleles
Source: PLoS One. 2013 Mar 5;8(3):e58352. doi: 10.1371/journal.pone.0058352 (PMC3589422; doi:10.1371/journal.pone.0058352)
Supplement: File S1 — List of the principle investigators in the International Multiple Sclerosis Genetic Consortium (IMSGC). (DOCX) [file pone.0058352.s004.docx]

List of the principle investigators in the IMSGC**:**

**International Multiple Sclerosis Genetic Consortium (IMSGC) membership**

Lisa Barcellos^1^, David Booth^2^, Manuel Comabella^3^, Alastair Compston^4^, Sandra D’Alfonso^5^, Philip De Jager^6^, Bertrand Fontaine^7^, An Goris^8^, David Hafler^9^, Jonathan Haines^10^, Hanne F. Harbo^11^, Stephen L. Hauser^12^, Clive Hawkins^13^, Bernhard Hemmer^14^, Jan Hillert^15^, Adrian Ivinson^16^, Ingrid Kockum^17^, Roland Martin^18^, Filippo Martinelli Boneschi^19^, Jacob L. McCauley^20^ Jorge Oksenberg^12^, Tomas Olsson^17^, Annette Oturai^21^, Nikolaos Patsopoulos^6^, Margaret Pericak-Vance^20^, Janna Saarela^22^, Stephen Sawcer^4^, Anne Spurkland^23^, Graeme Stewart^2^, Frauke Zipp^24^

^1^Genetic Epidemiology and Genomics Laboratory, Division of Epidemiology, School of Public Health, University of California, Berkeley, California, USA.

^2^ Westmead Millennium Institute, University of Sydney, New South Wales 2145, Australia.

^3^ Clinical Neuroinmunology Unit, Multiple Sclerosis Center of Catalonia (CEM-Cat), Vall d’Hebron University Hospital, Barcelona, Spain.

^4^ Department of Clinical Neurosciences, University of Cambridge, Addenbrooke’s Hospital, Cambridge, CB2 0QQ, UK

^5^ Department of Medical Sciences and Interdisciplinary Research Center of Autoimmune Diseases (IRCAD), University of Eastern, Piedmont, Novara, Italy

^6^ Department of Neurology, Brigham & Women’s Hospital and Harvard Medical School, Boston, MS, USA

^7^ INSERM UMR S 975 CRICM, UPMC, Département de neurologie Pitié-Salpêtrière, AP-HP, Paris, France

^8^Laboratory for Neuroimmunology, Section for Experimental Neurology, Katholieke Universiteit Leuven, Leuven, Belgium

^9^ Departments of Neurology and Immunobiology, Yale School of Medicine, New Haven, CT, USA.

^10^ Center for Human Genetics Research, Vanderbilt University Medical Center, 519 Light Hall, Nashville, Tennessee 37232, USA

^11^ Department of Neurology, Oslo University Hospital, Ullevål and University of Oslo, Oslo Norway

^12^ Department of Neurology, University of California San Francisco, San Francisco, USA

^13^ Keele University Medical School, Stoke-on-Trent ST4 7NY, UK

^14^ Klinik fur Neurologie, Klinikum rechts der Isar, Technische Universitat, Munchen, Germany

^15^ Department of Clinical Neurosciences, Center for Molecular Medicine, Karolinska Institutet, Karolinska University Hospital, Stockholm, Sweden.

^16^ Harvard NeuroDiscovery Center, Harvard Medical School, Boston, Massachusetts, USA.

^17^ Department of Clinical Neuroscience, Neuroimmunology Unit, Center for Molecular Medicine, Karolinska Institutet, Stockholm, Sweden

^18^ Institute for Neuroimmunology and Clinical MS Research (inims), Centre for Molecular Neurobiology, Falkenried 94, D-20251 Hamburg, Germany.

^19^ Department of Neurology, Institute of Experimental Neurology (INSPE), Division of Neuroscience, San Raffaele Scientific Institute, Milan, Italy

^20^ John P. HussmanInstitute for HumanGenomics and The Dr. John T Macdonald Foundation Department of Human Genetics, University of Miami, Miller School of Medicine, Miami, Florida , USA

^21^ Danish Multiple Sclerosis Center, University of Copenhagen and Department of Neurology, Rigshospitalet, Copenhagen, Denmark

^22^ Institute for Molecular Medicine Finland FIMM, University of Helsinki, Helsinki, Finland

^23^ Institute of Basal Medical Sciences, University of Oslo, Norway

^24^ Department of Neurology, University Medicine Mainz, Johannes Gutenberg University Mainz, Mainz, Germany.
